# Losartan improves clinical outcome in Camurati Engelmann Disease

**DOI:** 10.1186/1687-9856-2013-S1-O42

**Published:** 2013-10-03

**Authors:** Ahila Ayyavoo, Tim Cundy, José GB Derraik, Paul L Hofman

**Affiliations:** 1 Liggins Institute, University of Auckland, Auckland, New Zealand; 2 Greenlane Clinical Centre, Auckland District Health Board, Auckland, New Zealand

## 

We hypothesized that losartan would help in achieving clinical remission in CED (Camurati Engelmann Disease) patients by blocking TGFB1(transforming growth factor beta 1) with fewer side-effects than steroids.

CED characterised by progressive diaphyseal dysplasia is associated with debilitating bone pain in the limbs, muscle weakness, fatiguability and waddling gait.[1] Clinical manifestations are due to mutations in the TGFB1 gene leading to its over-expression and effect on bone. Losartan is an antagonist of TGFB1 and it slows the progress of aortic root dilatation inMarfan’s syndrome by blocking the over-expression of TGFB1.[2] Steroids which have long been used for treatment of CED and been linked to long term side effects including those on growth, blood pressure and spinal osteoporosis.

A 10 year old child with mutation is in exon 4, position C652T causing an R218C amino acid substitution on chromosome 19q13 had severe limitation of activity since 4 years of age due to pain in the limbs. She underwent a physical examination, a dual energy xray absorptiometry scan(DEXA), pain score and 6 minute walk test prior to the start of losartan with a repeat of the tests 9 and 17 months later. She is being treated with losartan at a dose of 0.75mg/kg/day. Table 1

**Table 1:** 

Age at analysis(years)	9.3	10.1	10.7
Cumulative pain score	9	1.75	0.25
6 minute walk test(metres)	171	405	414
DEXA weight(kgs)	17.42	17.01	20.01
Height(cms)	123.6	128.3	131.7
Fat(gms)	1702	1253	2693
Lean(gms)	14963	14957	16425
BMC(gms)	754.9	805	890.2
A/G ratio	0.29	0.23	0.39
Total body fat%	10.2	7.7	14.1
BMD gm/cm2	0.845	0.873	0.887

Losartan improves the quality of life in children with CED by reducing the bone pain along with improvement in their activity levels, fat & muscle mass, without major effects on growth, blood pressure and spinal osteoporosis.
